# Tools for Genomic and Transcriptomic Analysis of Microbes at Single-Cell Level

**DOI:** 10.3389/fmicb.2017.01831

**Published:** 2017-09-20

**Authors:** Zixi Chen, Lei Chen, Weiwen Zhang

**Affiliations:** ^1^Laboratory of Synthetic Microbiology, School of Chemical Engineering and Technology, Tianjin University Tianjin, China; ^2^Key Laboratory of Systems Bioengineering (Ministry of Education), Tianjin University Tianjin, China; ^3^SynBio Research Platform, Collaborative Innovation Center of Chemical Science and Engineering Tianjin, China; ^4^Center for Biosafety Research and Strategy, Tianjin University Tianjin, China

**Keywords:** single-cell analysis, microbes, heterogeneity, genomics, transcriptomics, next-generation sequencing

## Abstract

Microbiologists traditionally study population rather than individual cells, as it is generally assumed that the status of individual cells will be similar to that observed in the population. However, the recent studies have shown that the individual behavior of each single cell could be quite different from that of the whole population, suggesting the importance of extending traditional microbiology studies to single-cell level. With recent technological advances, such as flow cytometry, next-generation sequencing (NGS), and microspectroscopy, single-cell microbiology has greatly enhanced the understanding of individuality and heterogeneity of microbes in many biological systems. Notably, the application of multiple ‘omics’ in single-cell analysis has shed light on how individual cells perceive, respond, and adapt to the environment, how heterogeneity arises under external stress and finally determines the fate of the whole population, and how microbes survive under natural conditions. As single-cell analysis involves no axenic cultivation of target microorganism, it has also been demonstrated as a valuable tool for dissecting the microbial ‘dark matter.’ In this review, current state-of-the-art tools and methods for genomic and transcriptomic analysis of microbes at single-cell level were critically summarized, including single-cell isolation methods and experimental strategies of single-cell analysis with NGS. In addition, perspectives on the future trends of technology development in the field of single-cell analysis was also presented.

## Introduction

Microbiologists usually study microorganisms by deciphering their physiology, internal interactions, and even genetic information. Traditionally, these studies are all carried out at the population level, typically using millions to billions of cells for analysis in bulk, and assuming the status of individual cells is similar to that observed in the population. Although these results are, no doubt, informative, they often neglect any heterogeneity that is possibly present in the population. Meanwhile, the recent studies have shown that cell-to-cell heterogeneity at both cellular and molecular levels in isogenic population could be an order of magnitude greater than previously thought (Lidstrom and Meldrum, 2003), suggesting the importance of extending traditional microbiology studies to the single-cell level. It is now increasingly accepted that conclusions based on conventional average molecular or phenotypic measurements of a population could be biased, as the patterns of distinct sub-populations cannot be revealed (Wang et al., 2015).

Heterogeneities could result from either phenotypic difference between isogenic cells or genetic diversity at population level (Davis and Isberg, 2016). Mechanisms responsible for the cell-to-cell variation could be classified into four categories: stochastic gene expression, phenotypic plasticity, genotypic plasticity, and reversible genotypic variation (Roberfroid et al., 2016). While stochastic gene expression and phenotypic plasticity only lead to phenotypic differences, genotypic plasticity and reversible genotypic variation could introduce heterogeneity to an isogenic population at the genetic level. Stochastic gene expression widely exists in both prokaryotic and eukaryotic populations and is not exclusively driven by genomic information. Noise is one of the mechanisms of stochastic variability, which could be independent of environmental signals. This variation, either triggered by intrinsic or extrinsic noise, is usually unimodal. However, a unimodal noisiness of gene expression may trigger a bimodal behavior of downstream gene expression, leading to bimodality and bistability in a population (Dubnau and Losick, 2006; Veening et al., 2008). As different subpopulations co-exist, some individual cells may express genes that allow them to survive stresses prior to environmental changes. By using this mechanism, microbes could ensure that some individuals will survive under harsh conditions (Veening et al., 2008). Phenotypic plasticity is a kind of environmental-driven viability and could make it possible for cells to adapt to the fluctuations in the environment (Viney and Reece, 2013). An example is that in heterogeneous environmental conditions such as biofilm, isogenic cells could differentiate into various phenotypes and form several sub-populations for adapting to their local environmental conditions (van Gestel et al., 2015). Genotypic plasticity usually occurs in populations subjected to the experimental evolution. Driven by clonal evolution, clonal cells could evolve and finally result in genotypic diversification (Korona et al., 1994; Rainey and Travisano, 1998). Various mechanisms of genotypic diversification, such as clonal interference (Barrick and Lenski, 2013), niche construction, and niche partitioning (Barrick and Lenski, 2009), have been reported for both well-mixed and spatially structured environments (Roberfroid et al., 2016). As the final cause of variation, reversible genotypic variations are driven by random site-specific recombination, gene conversion, or epigenetic modification, leading to phase variations that play important roles in the virulence of some pathogens, and causing increased heterogeneity in the population (Davis and Isberg, 2016; Roberfroid et al., 2016).

Another major shortage of traditional microbiology approaches is the dependence on establishing laboratory culture for studying targeted microbes. Meanwhile, it is well-known that so far only a small number of microbial species in the biosphere could be cultivated successfully in the laboratory, leaving a great deal of microbial information untouched (Cardenas and Tiedje, 2008; Rinke et al., 2013). The hidden information, also known as microbial ‘dark matter,’ has drawn great interests recently and provides potential solutions for several critical issues, such as new drugs and antibiotics discovery (Ling et al., 2015), toxic chemicals degradation (Jiang et al., 2016), understanding pathogen virulence and disease mechanisms (Omsland et al., 2009), and revealing the human microbiome (Browne et al., 2016). Although obtaining axenic culture from natural isolates remains important, it is usually labor-intensive (Connon and Giovannoni, 2002), having a low success rate, and might be biased (Wu et al., 2009). In addition, comparing with the axenic cultures in the laboratory, microorganisms usually live in a more complex and barren environment in nature, making it unable to present the original state of microorganisms in the laboratory (Stewart, 2012). In recent years, many attempts have been employed for analyzing the microbe without axenic culture. For example, metagenomics and metatranscriptomics have been widely used for studying microbial community (Venter et al., 2004; Tringe et al., 2005; Mason et al., 2012, 2014; Meng et al., 2014). However, metagenomics and metatranscriptomics are not well-suited to reveal unambiguous information about the organization of discovered genes within genomes, evolutionary histories of specific organisms, and *in situ* interactions among organisms (Yoon et al., 2011; Stepanauskas, 2012). Genomic information, such as genome rearrangements, gene insertions, duplications and loss, is hard to obtain from metagenomic analysis since the assembled results could be mosaics of DNA from cells sharing high-homology regions but vary in genome-wide similarity (Stepanauskas, 2012).

Single-cell analysis can be effective for addressing these issues and providing better and in-depth understanding of the status of microbial cells. As it starts from only one cell, single-cell analysis could reveal information about individual cell without laboratory cultivation. With the help of high-throughput sequencing, it is possible to obtain functional genomics information of each single cell in its natural environment, so that its original genetic and functional status in a complex community can be revealed globally, quantitatively, and absolutely. Several reports using single-cell analysis have successfully revealed information like coexisting subpopulations, organismal interactions, and new metabolic pathways from uncultivated samples, which could hardly be obtained by traditional approaches (Marcy et al., 2007b; Hess et al., 2011; Siegl et al., 2011; Yoon et al., 2011; Martinez-Garcia et al., 2012a; Kashtan et al., 2014). In recent years, significant progress has been made to apply metagenomics and metatranscriptomics approaches to reveal the genetic information and gene expression patterns of cells in a population, and uncover microbial species and gene diversity in a community (Bowler et al., 2009). However, as metagenomics and metatranscriptomics could not reveal the information such as repetitive regions or strain heterogeneity comprehensively in a complex population, single-cell-based analysis has been proposed as a valuable supplement to the efficient identification of novel microbial species and the accurate interpretation of the metagenomics and metatranscriptomics results (Massana et al., 2014; Vannier et al., 2016; Ji et al., 2017). In this review, we summarize current state-of-the-art tools and methods for genomic and transcriptomic analysis of microbes at single-cell level, including single-cell isolation methods and experimental strategies of single-cell analysis with NGS, and provide some perspectives on the future trends of technology development in single-cell analysis field.

## Tools for Single-Cell Isolation

**FIGURE 1:**
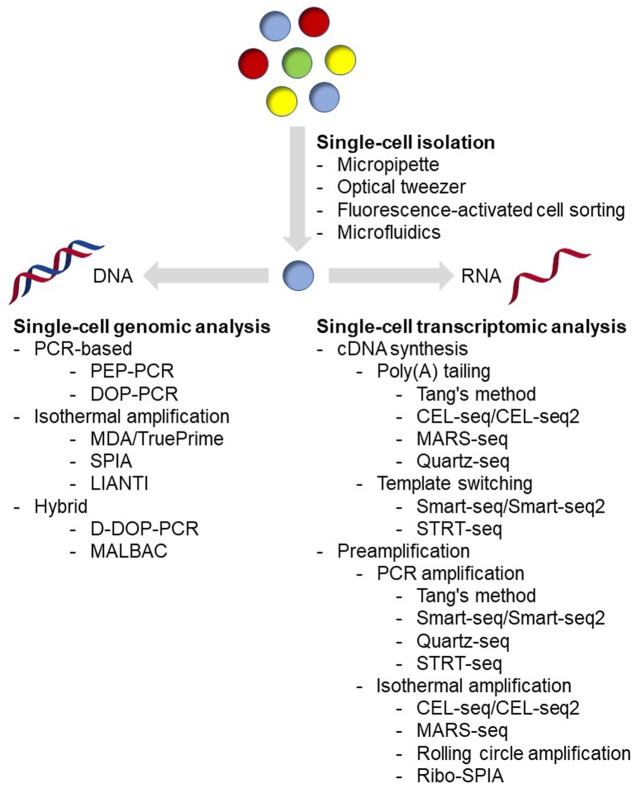
Overview of current single-cell analysis.

Single-cell isolation is the very first step in the single-cell analysis process (**Figure 1**). The major challenge of this step is: how to isolate cells of interest accurately in a high-throughput manner and without causing any genetic or physiological change to the target cells. Basically, methods being applied for microbial single-cell isolation could be classified as two principal approaches: micromanipulation and random encapsulation (Blainey, 2013). Micromanipulation methods, including micropipette and optical tweezer approaches, are carried out under high-resolution microscope. These methods offer a great confidence that every single cell can be observed, captured and delivered to the next step. Traditional micropipette method could be easily applied in any laboratory on an inverted microscope with mechanical liquid handling. Although very labor-consuming and low-throughput, approximately in the order of 50 cells/h and person (Picelli, 2016), it is the first choice if only a small number of cells are required for the next step analysis (Qi et al., 2014, 2016; Wang et al., 2015). In addition, commercial robotic manipulation system for automated single-cell selection has also been developed and applied for microbial single-cell analysis (Anis et al., 2008; Merza et al., 2009; Gao et al., 2011; Banerjee et al., 2014), making it possible for relatively high-throughput single-cell isolation. Optical tweezer approaches are implemented by tightly focusing a laser beam for trapping cells in solution (Ashkin et al., 1986, 1987; Ashkin and Dziedzic, 1987). Usually, by using near-infrared wavelengths of light, cells could be easily handled without any harm (Neuman et al., 1999; Ericsson et al., 2000). This method has been successfully applied in many microbial isolation experiments, including filamentous bacteria (Pamp et al., 2012) and even virus (Ashkin and Dziedzic, 1987). More detailed reviews of this method have been published recently (Moffitt et al., 2008; Hashemi Shabestari et al., 2017), interested readers could refer to these articles.

Flow cytometry and microfluidic device are the most widely used random encapsulation approaches in recent years. Flow cytometry and FACS have a much higher throughput, and have been demonstrated as an effective platform for single-cell analysis in microbial cells (Raghunathan et al., 2005; Stepanauskas and Sieracki, 2007; Woyke et al., 2009; Swan et al., 2011; Martinez-Garcia et al., 2012b; Field et al., 2015). Although FACS is fundamentally based on random encapsulation, flow cytometers can monitor several parameters of individuals, which means single cells can be sorted according to their size, morphology, spontaneous fluorescence, fluorescence-labeled antibodies and staining dyes simultaneously, making it possible to sort even rare cell types. In addition, it is easy to sort single cells directly into 96- and 384-well plates using commercial instruments, which means single-cell analysis workflow could be entirely performed using automated liquid handling robots. However, cells are typically subjected to physical stresses during the sorting, such as fluidic pressure, laser beam, electrostatic charges, voltage fields, and collisions with container surfaces, which could significantly affect the cell physiology and even the recovery rate during the cultivation (Marie et al., 2017). In the case when the sorted cells are used for gene expression or transcriptome analysis, proper RNA protectant needs to be added (Qi et al., 2014, 2016; Wang et al., 2015); while in the case when the sorted cells are used for clonal cultivation, extra efforts to carefully optimize the cultivation conditions are necessary in order to maximize the success rates (Marie et al., 2017). Under the conception of ‘Lab-on-a-chip,’ microfluidic devices have become the most popular method for single-cell isolation. With these devices, researchers could integrate single-cell analysis process from cell isolation to sequencing library preparation in only a coin-sized microchip, which could be either purchased from commercial manufacturers or designed and fabricated using materials such as PDMS in the laboratory. Combining with detection technologies, such as fluorescence spectroscopy (Wolff et al., 2003) or raman spectroscopy (Song et al., 2016), microfluidic devices could perform specific sorting while encapsulating cells with reagents for cell lysis and sequencing library preparation at nanoliter volume with high-throughput (Klein et al., 2015). Comparing with traditional tube-based reactions, microfluidic devices require few manual liquid handling, leading to a significant decrease in contamination and less variations among samples (de Bourcy et al., 2014). Notably, less contamination with nanoliter reaction volume means a higher concentration of substrates, resulting in better uniformity amplification (Fu et al., 2015; Leung et al., 2016). In addition, comparing with other methods, microfluidic devices cause less physical stresses to cells, leading to more accurate physiological analysis and high success rates of further cultivation analysis (Jiang et al., 2016; Kim et al., 2017; Song et al., 2017; Zhang et al., 2017). In general, both flow cytometry and microfluidic devices could provide high-throughput and accurate single-cell sorting. Flow cytometers could monitor multiple parameters and are capable for rare cell detection and sorting, but are usually expensive and require skilled operators. Before sorting, it is also necessary to prepare a sterile system for flow cytometers to prevent contamination. By contrast, microfluidic devices can be designed and made in the laboratory. They can be disposable in order to minimize contamination, and are easy to operate. Besides single-cell sorting, microfluidic devices could offer integrative single-cell analysis including cell culture and tracking (Yu et al., 2017), digital PCR (Ottesen et al., 2006), and sequencing library preparation (Hosokawa et al., 2017; Lan et al., 2017). With several advantages mentioned above, in recent years, microfluidic devices tended to be used as an analytic platform rather than just an isolation method for single-cell analysis (Marshall et al., 2012; Zhang et al., 2015, 2017; Jiang et al., 2016; Haliburton et al., 2017; Hosokawa et al., 2017; Kim et al., 2017; Lan et al., 2017; Shahi et al., 2017; Song et al., 2017). Several detailed reviews have been published recently on microfluidic devices (Wen et al., 2016; Caen et al., 2017; Prakadan et al., 2017; Xi et al., 2017), and interested readers could refer to these articles.

## Tools for Genomic Analysis at Single-Cell Level

A single microbial cell usually contains picogram to femtogram level of genomic DNA (Kim et al., 2017). Sequencing technologies, up to now, are still unable to sequence such a low amount of nucleic acids directly without any amplification. Therefore, researches have been applying WGA methods since 1990 (Lichter et al., 1990). However, as amplification is conducted using DNA polymerases, the amplified products could contain genetic information of the original cell as well as some artifacts, such as genome fragment loss, amplification bias, mutations, and chimeras. Over the past 20 years, WGA methods have been optimized with substantial progress, including less contamination and better amplification performance (Blainey, 2013). In general, amplification methods could be classified into three categories: pure PCR-based amplification, isothermal amplification, and hybrid methods (Gawad et al., 2016).

Pure PCR-based WGA methods are the primary methods at early stage in the single-cell genomic analysis. Early approaches with specific primers, such as linker-adapter (also known as ligation-anchored) PCR (LA-PCR) (Troutt et al., 1992; Klein et al., 1999) and IRS-PCR (Lengauer et al., 1990; Lichter et al., 1990), require ligation reaction or prior knowledge of the target sequence. Later, methods with random primers, including primer extension pre-amplification PCR (PEP-PCR) (Hubert et al., 1992) and DOP-PCR (Telenius et al., 1992) were introduced. As the most representative method in this category, DOP-PCR typically contains two stages, with the first facilitating random primer extension on the template genome DNA and the second favoring amplicon replication with specific primer (Telenius et al., 1992).

The second category of WGA is isothermal amplification, which was first reported in Dean et al. (2001); Zhang et al. (2001) and has been demonstrated as a powerful tool in microbial single-cell genomic analysis, especially with MDA (Lasken, 2012). Unlike PCR-based methods, isothermal amplification methods use polymerases with strong strand displacement activity, such as φ29 polymerase, and 6-mer 3′-protected random primers for isothermal extension (Dean et al., 2001; Zhang et al., 2001). During extension, polymerase creates and displaces synthesized products from single-stranded DNA template, and the displaced DNA is the template for further priming and synthesis (Dean et al., 2001; Zhang et al., 2001). Compared with PCR-based methods, MDA shows higher genome coverage, lower error rates and much longer extension length over 10,000 nt (Blanco et al., 1989; de Bourcy et al., 2014). However, the loci amplified first are typically found to be overrepresented, indicating non-uniformity of MDA (de Bourcy et al., 2014). Recently, a novel primer-free method called TruePrime was reported and has been successfully used for the amplification of genomic DNA from single human HEK293 cells (Picher et al., 2016). In this method, an enzyme called *Tth*PrimPol, which has a wide range of template specificity, serves as primase for φ29 polymerase mediated MDA. During the reaction, *Tth*PrimPol binds to the denatured DNA and synthesizes short DNA primers. The DNA primers are recognized and extended by φ29 polymerase. Then, *Tth*PrimPol catalyzes new rounds of priming on the elongated single-strand DNA, followed by further rounds of strand-displacement synthesis and resulting in exponential amplification (Picher et al., 2016). Another isothermal amplification method, called SPIA, could achieve linear amplification under isothermal conditions by using a specific DNA/RNA hybrid primer, together with RNase H and a strand-displacing DNA polymerase (Kurn et al., 2005). In SPIA method, strand-displacement only occurs at the DNA/RNA hybrid primer site of the amplicons, preventing the exponential amplification in MDA. Recently, a new method called LIANTI was reported (Chen et al., 2017). As an isothermal amplification method, this approach depends on RNA polymerase but not DNA polymerase for linear amplification. In this method, genomic DNA from a single cell was fragmented and tagged by Tn5 transposon with a T7 promoter, then linear amplified with T7 RNA polymerase, and finally converted to DNA by reverse transcription for further library preparation (Chen et al., 2017).

Two similar hybrid methods, displacement DOP-PCR (D-DOP-PCR, also known as PicoPLEX or GenomePlex) (Langmore, 2002) and MALBAC (Lu et al., 2012; Zong et al., 2012), were recently developed to overcome the low coverage of PCR-based methods and the non-uniformity of MDA. These two methods both use isothermal amplification followed by PCR amplification, but different primers for extension. D-DOP-PCR uses degenerated primers in the first step adding an anchor sequence with isothermal amplification and then using PCR amplification for the second step (Langmore, 2002). MALBAC, however, uses a random primer with a designed anchor which could promote looping of the isothermal amplification products to prevent further amplification before the second PCR step, suggesting a more uniform amplification (Lu et al., 2012; Zong et al., 2012).

In practice, isothermal and hybrid methods are currently the most commonly used approaches, as they show better performance comparing with pure PCR-based methods. Several groups have compared these methods using both microbial and mammalian cells (Chen et al., 2014; de Bourcy et al., 2014; Deleye et al., 2015; Hou et al., 2015; Ning et al., 2015). These reports have drawn similar conclusions that MDA has significantly higher genome coverage breadth and lower false-positive rates, while hybrid methods demonstrate better coverage uniformity (Chen et al., 2014; de Bourcy et al., 2014; Deleye et al., 2015; Hou et al., 2015; Ning et al., 2015). For example, one report showed that MDA has better coverage breadth than MALBAC (84% vs. 52%), resulting in higher detection rates of SNVs (88% vs. 52%) in human cells (Hou et al., 2015). Another report showed that hybrid methods has better coverage uniformity than MDA, suggesting that hybrid methods have better performance than MDA in detecting CNVs (Ning et al., 2015). In the report, the researchers also found that MALBAC tended to over-amplify genomic regions with a high-GC content (Ning et al., 2015). The average GC content of amplified DNA using GenomePlex (41.6%) was very close to the reference genome (41.9%), while the average GC contents of amplified DNA regions by MDA and MALBAC were 43.4 and 46.6%, respectively (Ning et al., 2015). However, after a GC-correction, the correlation of read abundance between MALBAC and bulk-cell samples (*R*^2^ = 0.53) was nearly the same as GenomePlex (*R*^2^ = 0.56), while MDA gave a very poor correlation (*R*^2^ = 0.02) (Ning et al., 2015). The TruePrime method was reported to have better coverage uniformity than the primer-based MDA, leading to an improved CNV detection accuracy, thus an advantage over the traditional primer-based MDA protocol (Picher et al., 2016). In addition, by using human genomic DNA as input, TruePrime could amplify as low as 1 fg DNA, which is about 100-fold more sensitive than the primer-based MDA (Picher et al., 2016). This superior sensitivity could be very valuable for microbial single-cell genomic analysis, as most microbes are much smaller and contain less DNA than eukaryotic cells. Notably, the most recent LIANTI method exhibited significantly improved amplification uniformity and genome coverage over the previous methods on all scales, and was capable for both high accuracy of CNV detection and low SNV false-positive rate (Chen et al., 2017). As new invented approaches, both TruePrime and LIANTI have the potential but still need more evaluation to demonstrate their performance on microbial single-cell genomic analysis. In conclusion, there is no clear winner in performance between MDA and hybrid methods yet, and researchers should choose methods depending on the metric of their interest (Gawad et al., 2016). As microbial single-cell analysis usually focuses on elucidating the genomic information of the microbial ‘dark matter,’ genome coverage is the key to be concerned. Therefore, MDA method has been far more widely used for microbial single-cell analysis rather than the others.

Besides amplification methods, previous reports also found that by using microfluidic devices, microbial single-cell analysis could obtain a better performance comparing with the traditional tube-based approach (de Bourcy et al., 2014). With higher mapping ratio and better repeatability, microfluidic devices could also reduce the contamination especially from the experiment operator (de Bourcy et al., 2014). Recently, two groups independently reported high-throughput microbial single-cell analysis protocols based on self-designed microfluidic devices (Hosokawa et al., 2017; Lan et al., 2017). These two protocols shared some similarities in single microbe encapsulation and lysis protocols. However, one protocol involved sorting the positive amplification droplets and re-amplification of the DNA for further analysis by NGS and qPCR (Hosokawa et al., 2017), while the other protocol used a strategy of labeling DNA fragments from the same cell with a barcode, and then pooling and sequencing of the barcoded fragments of all cells (Lan et al., 2017). These protocols could provide reliable pipelines for analyzing 10s of 1000s of single microbial cells within a couple of hours with a comparable performance to the conventional techniques. In conclusion, with further improvements on both amplification methods and microfluidic devices, microbial single-cell genomic analysis will be more efficient, reliable, and convenient in the near future.

## Tools for Transcriptomic Analysis at Single-Cell Level

Prior to whole-genome transcriptomic analysis, relative quantification methodologies have been developed to measure expression of small number of genes at single-cell level. For example, methods using fluorescent reporter proteins coupling with high-throughput data acquisition approaches such as flow cytometry have been widely applied for detecting gene expression heterogeneities within the microbial population (Taniguchi et al., 2010; Roberfroid et al., 2016). In addition, methods using RT-qPCR for detecting gene expression in single cells have also been reported and successfully applied to several types of microbes for heterogeneity analysis (Gao et al., 2011; Shi et al., 2013; Qi et al., 2014, 2016; Wang et al., 2015; Thompson et al., 2017; Turkarslan et al., 2017). However, these methods could only reveal gene expression patterns of a very limited number of genes, while not able to uncover global information in a cell. Moreover, application of such approaches typically requires genetic engineering tools and genomic information of the target microorganisms, limiting the application to only model organisms.

Global transcriptomic analysis could circumvent the above drawbacks and even possible for unknown species without genome information using *de novo* NGS approach. Compared to genomic analysis, transcriptomic analysis for microbes at single-cell level is much more challenging for several reasons. First, microbial cells usually contain picogram to femtogram level RNA molecules (de Bekker et al., 2011; Kang et al., 2011; Wang et al., 2015), while mammalian cells could have up to nanogram level RNA molecules (Picelli, 2016). Besides the low-RNA content, RNA molecules of prokaryotic cells are less stable than DNA and could be degraded by ribonucleases that are widely existing and hard to be deactivated. Moreover, rRNA and tRNA molecules usually represent over 90% of total RNA, but offer limited biological information and should be excluded in the amplification process, as most researches focus on mRNA and other rare molecules. With a complicated cell wall, harsher conditions are typically required to lyze a microbial cell, which may lead to damage or loss of RNA, and accuracy and efficiency of the downstream transcriptomic analysis (Khan and Yadav, 2004; Hall et al., 2013; Heera et al., 2015; He et al., 2016). More importantly, unlike genomic analysis, in which the methods for mammalian cells could be also readily applied to prokaryotic microbes, not all methods for mammalian single-cell transcriptomic analysis could be used to microbes. This is simply because of the structure differences of mRNA molecules between eukaryotic and prokaryotic cells. Currently, most of the mammalian single-cell transcriptomic analysis approaches use oligo(dT) primers in the first cDNA synthesis step. This is based on the 3′ poly(A) structure of mRNA molecules from eukaryotic cells, which makes them easier to be enriched from rRNA and tRNA. However, mRNA molecules from prokaryotic cells usually lack the poly(A) tail, and require random primers for cDNA synthesis. By using random primers, both rRNA and tRNA will also be included in the resulting transcriptome library, thus being sequenced together, leading to a low coverage of the target mRNA. In addition, application of random primers for cDNA synthesis causes losses of 3′ sequence information, as they are usually unable to obtain the full-length transcripts. Hence, so far only a few reports on prokaryotic single-cell transcriptomic analysis have been reported (Kang et al., 2011, 2015; Wang et al., 2015). Even for eukaryotic microbes that could be analyzed with well-developed approaches for mammalian cells, only one report analyzing single-cell transcriptomics of neighboring hyphae of *Aspergillus niger* was reported (de Bekker et al., 2011). Concerning this circumstance, we summarized below all the state-of-the-art tools in single-cell transcriptomic analysis and discussed possibilities for their microbial applications, especially for prokaryotic microorganisms.

To our knowledge, the earliest study of single-cell transcriptomics was reported in Eberwine et al. (1992). In this work, mRNA molecules from single-living neurons were reverse transcribed to cDNA using oligo(dT)-T7 primer. Then, the synthesized double-stranded cDNA molecules with T7 promoter were used as templates for IVT with T7 RNA polymerase for producing amplified RNA. Next, the amplified RNA molecules were used as templates for the second turn of reverse transcription. After this process, over a million-fold amplification of the original RNA was achieved. Although this report only used ISH for accessing gene expression, it reveals the possibility about analyzing gene expression at a single-cell level. Based on the concept of this study, several studies have successfully analyzed the whole transcriptome of single mammalian cells (Morris et al., 2011; Hashimshony et al., 2012, 2016; Jaitin et al., 2014).

In the past decade, several new approaches were developed, leading to a tremendous progress in mammalian single-cell RNA-seq (Tang et al., 2009, 2010; Islam et al., 2011, 2012; Goetz and Trimarchi, 2012; Hashimshony et al., 2012, 2016; Picelli et al., 2013, 2014; Sasagawa et al., 2013; Jaitin et al., 2014; Soumillon et al., 2014; Fan H.C. et al., 2015; Klein et al., 2015; Macosko et al., 2015). The most widely used single-cell RNA-seq methods are characterized in **Table 1**. As these methods have been well reviewed in several excellent articles (Saliba et al., 2014; Chen et al., 2015; Kolodziejczyk et al., 2015; Picelli, 2016), we will focus only on some newly developed methods here. Among the methods listed in **Table 1**, Smart-seq/Smart-seq2 and Quartz-seq use a method called ‘template switch’ for the second strand cDNA synthesis, generating full-length double-stranded cDNA comparing with the others (Goetz and Trimarchi, 2012; Picelli et al., 2013, 2014; Sasagawa et al., 2013). Smart-seq, CEL-seq2 and STRT-seq are compatible with Fluidigm C1 Single-Cell Auto Prep system, which is an automated platform and captured using integrated fluidic circuits (Ziegenhain et al., 2017). For amplification types, Tang’s method, Smart-seq/Smart-seq2, Quartz-seq, and STRT-seq are all based on PCR amplification, while CEL-seq/CEL-seq2 and MARS-seq are based on IVT. The advantage of IVT is that the amplification efficiency is sequence independent. However, as it requires a second time of reverse transcription, there is 3′ coverage bias of the sequencing results (Kolodziejczyk et al., 2015). When choosing an appropriate single-cell RNA-seq method, transcript coverage, strand specificity, position bias, and UMI compatibility should be concerned depending on the purpose of the research (**Table 1**). For example, full-length RNA-seq methods such as Smart-seq/Smart-Seq2, and Quartz-seq could sequence the transcripts in their entirety, and thus are suggested for *de novo* sequencing and the detection of SNPs and mutations. However, these methods are not compatible with strand-specific protocol and UMI. Methods such as STRT-seq, CEL-seq/CEL-seq2, MARS-seq, and Drop-seq are all compatible with strand-specific protocol and UMI, although they tend to be 5′ or 3′ end biased. While UMI approach in single-cell sequencing could reduce amplification noise and provide more accurate expression quantification, strand-specific sequencing could provide more information for antisense transcript discovery, genome annotation, and expression profiling. In conclusion, current single-cell RNA-seq methods are still facing a trade-off between coverage and uniformity (Picelli, 2016). Notably, none of these methods has been evaluated in eukaryotic microbes, suggesting further optimization and development are needed for microbial cells. Recently, a systematically evaluation of six prominent single-cell RNA-seq methods has been reported (Ziegenhain et al., 2017), and the results indicated that Smart-seq2 had the best coverage because of its full-length synthesis ability. However, as Smart-seq2 is incompatible with UMIs, all methods using UMIs have less amplification noise. In addition, power simulations showed that Drop-seq is more cost-efficient for analyzing a large number of cells, while Smart-seq2, MARS-seq, and SCRB-seq are more efficient with the analysis of a small number of cells (Ziegenhain et al., 2017).

**Table 1:** Characteristics of several widely used single-cell RNA-seq methods.

Name	Transcript coverage	Position bias	Strand specificity	UMI compatible	Key reference
Tang’s method	Nearly full-length	Strongly 3′	No	No	Tang et al., 2009, 2010
Quartz-seq	Full-length	Weakly 3′	No	No	Sasagawa et al., 2013
Smart-seq/Smart-seq2	Full-length	Weakly 3′	No	No	Goetz and Trimarchi, 2012; Picelli et al., 2013, 2014
STRT-seq	5′ only	5′ only	Yes	Yes	Islam et al., 2011, 2012
CEL-seq/CEL-seq2	3′ only	3′ only	Yes	Yes	Hashimshony et al., 2012, 2016
MARS-seq	3′ only	3′ only	Yes	Yes	Jaitin et al., 2014
SCRB-seq	3′ only	3′ only	Yes	Yes	Soumillon et al., 2014
Drop-seq/InDrop	3′ only	3′ only	Yes	Yes	Klein et al., 2015; Macosko et al., 2015
Cyto-seq	Pre-defined genes only	3′ only	Yes	Yes	Fan H.C. et al., 2015

Besides the above methods, several new methods have also been developed recently. Some of them have already been utilized for single-cell RNA-seq in prokaryotic cells (Kang et al., 2011, 2015; Wang et al., 2015). The first case of single-cell microbial transcriptomic analysis, to our knowledge, was reported in Kang et al. (2011). In this report, transcriptome of single bacterium *Burkholderia thailandensis* was analyzed using microarray through amplification of RNA molecules by rolling circle amplification. In this report, bacterial cells were first lysed with Triton X-100 and lysozyme, and then the lysate was used for direct cDNA synthesis with random primers. After genomic DNA degradation, single-stranded cDNA molecules were self-ligated and then used as the template for multiply primed rolling circle amplification using φ29 polymerase with random primers. The result showed low fold-change bias and only less than 6% drop-outs with no contamination. In addition, this method also preferred an optional rRNA/tRNA elimination step for deep sequencing. By using 5′-phosphate-dependent exonuclease, rRNA and tRNA molecules, which have the 5′-phosphate structure, will be specifically degraded, leaving the mRNA molecules which lacking the 5′-phosphate structure for the next cDNA synthesis step. This is also the only report we could find, which has successfully depleted rRNA from single microbial cells, indicating the requirements for further innovation of other effective rRNA depletion and mRNA enrichment methods for microbes. Later in the same year, another approach using Ribo-SPIA method, that is, derived from SPIA method for amplification, has successfully been employed to analyze transcriptomics of neighboring hyphae of the eukaryotic fungus *A. niger* using microarray (de Bekker et al., 2011). In this article, total RNA from different 5 hyphal tips were isolated using a column based kit and amplified using the WT-Ovation One-Direct RNA Amplification System (Nugen) with both oligo(dT) and random primers. Microarray analysis resulted in a present call for 4–7% of the *A. niger* genes, of which 12% showed heterogeneous RNA levels, indicating the feasibility of using this method for microbial transcriptomic analysis. In another study with prokaryotic cells, Wang et al. (2015) successfully conducted single-cell RNA-seq in single cyanobacterium *Synechocystis* sp. PCC 6803 cells with Ribo-SPIA method. To determine the heterogeneity upon environmental stress, this method was applied to *Synechocystis* single cells at 24 and 72 h after nitrogen starvation treatment. With up to 98% of all putative *Synechocystis* genes identified in single cells, a possible increasing gene-expression heterogeneity from 24 to 72 h after nitrogen starvation stress was also found, indicating the method could achieve good identification of the transcripts in single bacterial cells (Wang et al., 2015). More recently, a technology for targeted depletion of abundant transcripts was developed by Nugen (Armour et al., 2015). Unlike the exonuclease-based depletion method that Kang et al. (2011) reported, this method depleted the unwanted sequences after cDNA synthesis using probes that target unwanted sequences. However, the information of the unwanted sequences is required, making it impossible for *de novo* single-cell RNA-seq. SUPeR-seq (Fan X. et al., 2015) is another method to sequence both polyadenylated and non-polyadenylated RNAs, suggesting its possible application to prokaryotic microbes. This method shares some similarities to Tang’s method (Tang et al., 2009, 2010), but used a primer containing an anchor sequence (AnchorX), 15-mer dT sequence and 6-mer random sequence for simultaneous detection of both polyadenylated and non-polyadenylated RNA molecules and synthesizing the first strand cDNA. After poly(A) tailing for the first strand cDNA, a primer containing another anchor sequence (AnchorY) and 24-mer dT sequence was used for second strand cDNA synthesis, and then the double-stranded cDNA molecules were amplified by PCR using AnchorX and AnchorY primers. With this approach, the researchers discovered 2891 circRNAs in mouse preimplantation embryos. Like other methods using random primers, rRNA could not be excluded with this method. However, this method provides another possible method for single-cell RNA-seq in prokaryotic microbes, especially with the rRNA depletion methods mentioned above.

## Future Perspectives

Current genomic and transcriptomic analysis of single microbial cells share several similar challenges. Cell lysis is a major challenge for single-cell analysis. As microbes typically contain complicated structure of cell walls, appropriate lysis strategies need to be chosen carefully without damaging the DNA/RNA inside. In addition, in the case if the lysate is directly used for amplification without purification, the lysis condition should also be carefully optimized to minimize the influence of lysis related reagents to the downstream reactions. Alternatively, a method called FluidFM might be a promising approach for DNA/RNA isolation from microbial cells, as it used a ‘nanosyringe’ to extract cytoplasmic and nucleoplasmic fractions from single live cells rather than lysis the cell (Meister et al., 2009; Guillaume-Gentil et al., 2016). Contamination is another key challenge in single-cell analysis. As low-input and high-fold amplification are required for sequencing, single-cell analysis is very sensitive to contamination, either from the laboratory environment or reagents and instruments used for sample preparation. Several approaches have been applied to minimize contamination, including reducing the reaction volume of lysis and amplification reaction to nanoliter scale in a sealed, disposable microfluidic device (Marcy et al., 2007a,b), using UV exposure to inactivate contaminates in reagents (Zhang et al., 2006; Woyke et al., 2011), and disposable plasticware produced from virgin materials (Blainey and Quake, 2011). Another challenge for microbial single-cell analysis is the ultra-low nuclei acids content in a single microbial cell. Current microbial single-cell sequencing methods were all modified from those developed for mammalian cells, as they contain more nuclei acids. While using these methods in microbial cells, nuclei acids template could be a 1000-fold less than using a mammalian cell. With a much lower concentration of templates, the amplification process could be more sensitive to any contamination and non-specific amplification. In addition, less input may also challenge the sensitivity of the polymerase used for the amplification process (Picher et al., 2016). Using microfluidic devices for amplification could significantly solve these problems (de Bourcy et al., 2014). Moreover, the low input also influences the uniformity of the amplification. Even for single-cell analysis of mammalian cells, the amplification uniformity is still not comparable with that at the bulk-cell level. Therefore, the sequencing depth could be a critical factor to ensure good genome coverage, especially for unculturable microbes with unknown genome sizes.

Current single-cell sequencing methods all require amplification of DNA/RNA from a single cell for NGS sequencing, which will inevitably introduce bias and loss. Recently, new sequencing platforms such as true single molecule sequencing (tSMS, Helicos, now SeqLL), SMRT sequencing (PacBio), and nanopore sequencing (Oxford Nanopore) could sequence DNA/RNA molecules at single-molecule level and prove to be possible to sequence DNA/RNA molecules directly from bulk-cells without pre-amplification (Ozsolak et al., 2009; Ozsolak and Milos, 2011; Coupland et al., 2012; Ayub et al., 2013). Although directly sequencing a single cell without pre-amplification is still challenging, further innovation of these new technologies and sequencing platforms could eventually make it possible for single-cell analysis without any amplification.

## Conclusion

As a rapidly growing field, single-cell analysis plays a significant role in extending our understanding of microorganisms by revealing how individual cells perceive, respond and adapt to the environment, and determine the fate of the whole population. The key drivers of new technology for single-cell analysis will be advancement in throughput, integration of isolation and amplification, and integrated analysis with multiple ‘omics.’ Even with many challenges still ahead, we believe that this field will receive a tremendous boost with progress of several related fields, such as microfluidic devices and new sequencing platforms.

## Author Contributions

WZ envisioned this project. ZC, LC, and WZ wrote the manuscript. All authors have read and agreed on the manuscript.

## Conflict of Interest Statement

The authors declare that the research was conducted in the absence of any commercial or financial relationships that could be construed as a potential conflict of interest.

## Abbreviations

CNV: copy-number variation
D-DOP-PCR: displacement DOP-PCR
DOP-PCR: degenerate oligonucleotide-primed PCR
FACS: fluorescence-activated cell sorting
FluidFM: fluidic force microscope
IPS-PCR: interspersed repetitive sequence PCR
ISH: *in situ* hybridization
IVT: *in vitro* transcription
LA-PCR: linker-adapter or ligation-anchored PCR
LIANTI: linear amplification via transposon insertion
MALBAC: multiple annealing and looping-based amplification cycles
MDA: multiple displacement amplification
NGS: next-generation sequencing
PDMS: polydimethylsiloxane
PEP-PCR: preamplification PCR
poly(A): polyadenylated
RNA-seq: RNA sequencing
SMRT: single molecule real-time
SNV: single nucleotide variant
SPIA: single primer isothermal amplification
tSMS: true single molecule sequencing
UMIs: unique molecular identifiers
WGA: whole genomic amplification.
